# Curative intent treatment of late presented extragastrointestinal stromal tumor: two identical case reports with literature review

**DOI:** 10.1093/jscr/rjab220

**Published:** 2021-06-04

**Authors:** Dhruba Kadel, Shashinda Bhuju, Bikash Raj Thapa, Sanat Chalise, Sandeep Kumar Sah

**Affiliations:** Department of General Surgery, Scheer Memorial Adventist Hospital, Banepa, Nepal; Department of General Surgery, Scheer Memorial Adventist Hospital, Banepa, Nepal; Department of Radiology, National Academy of Medical Sciences, National Trauma Center, Kathmandu, Nepal; Department of Pathology, Kathmandu Medical College Teaching Hospital, Kathmandu, Nepal; Department of General Surgery, Scheer Memorial Adventist Hospital, Banepa, Nepal

## Abstract

Gastrointestinal stromal tumors (GISTs) occurring outside the gastrointestinal tract are known as extragastrointestinal stromal tumors (EGIST). They share some common histopathologic and molecular characteristics. This report describes two female patients who were suspected of having a mesenteric GIST, but opted for surveillance rather than definitive treatment. Upon reassessment, both patients demonstrated increased tumor mass with no evidence of distant metastasis. The intraoperative findings confirmed the conclusion of clinical and imaging studies performed preoperatively and radical excisions were performed. Histopathological examination (spindle cell neoplasm) and immunohistochemistry (CD117) confirmed EGIST. Both patients underwent Imatinib therapy following surgery with no evidence of disease recurrence or metastasis upon follow up. Although sharing histologic features with GIST, EGIST frequently demonstrates distinct characteristics that facilitate the proper diagnosis and management of EGIST. Since it is a rare and aggressive disease with a poor outcome, early detection and curative surgical resection remains the mainstay of treatment.

## INTRODUCTION

Gastrointestinal stromal tumors (GISTs) comprise only 0.1–3% of all GI neoplasia, however, they represent the most common mesenchymal tumors accounting for >80% [1]. Because of their distinct morphologic and immunophenotypic profile compared with tumors arising from smooth muscle in other parts of body, GISTs are now recognized as a separate subgroup of mesenchymal tumors [2]. Extragastrointestinal stromal tumors (EGIST) are rarely reported tumors originating outside the gastrointestinal (GI) tract, but they are histologically similar to their GI counterparts. Although GISTs can metastasize to intra-abdominal organs such as the mesentery and omentum, EGISTs have been recognized as a distinct entity as they show some unique characteristics. As opposed to GISTs, which originate from interstitial cells of cajal, some researchers have pointed out that EGISTs originate from cajal-like cells or pluripotent stem cells located outside the GI tract [3, 4]. But positive staining for CD117 is still the gold standard criteria for diagnosis of both entities and the current recommendation of standard treatment is similar for both GIST and EGIST i.e. en bloc resection of the tumor with negative margins followed by adjuvant imatinib [5]. However, the clinical, radiological and histological features of EGIST are still largely unknown [6]. Moreover, the role of imatinib in the treatment of EGIST is still controversial [3, 4].

## CASE PRESENTATION-1

**Figure 1:**
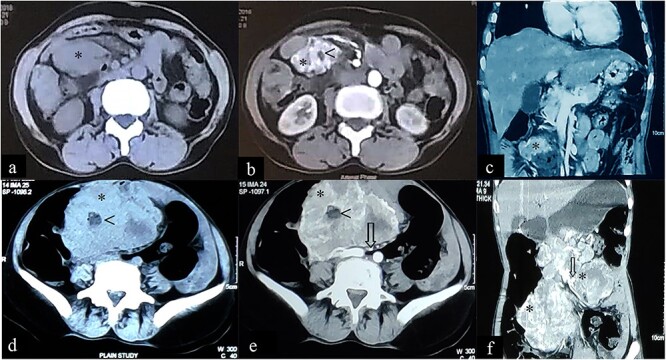
Case 1: CT imaging of extragastrointestinal stromal tumor. (**a**) Non-contrast axial, (**b**, **c**) contrast enhanced axial and coronal reformatted CT images of abdomen demonstrate heterogeneously enhancing confluent mass (*) in peritoneal cavity anterior to the iliac vessels with central area of necrosis (<) peripherally displacing mesenteric vessels (arrow) and bowel. (**d**–**f**) Non-contrast axial, contrast enhanced axial and coronal reformatted CT images of abdomen of same patient after 1-year follow-up showing increased interval size of the lesion (*).

A 62-year-old female agricultural worker, presented with complaints of gradual abdominal distension over the past 2 years, associated with mild to moderate abdominal pain (exacerbated by meals), anorexia and gradual weight loss (~15-kg weight loss in 2 years). She was first seen at another hospital 18-month ago where contrast enhanced computed tomography (CECT) of abdomen revealed two heterogeneously enhancing exophytic lesions; one in the second part of the duodenum (43 mm × 36 mm) and the second in the right lumbar region (50 mm × 34 mm). The radiologic interpretation suggested EGIST (Fig. 1a–c), but the patient elected to not undergo any further workup or treatment.

The patient presented to our outpatient clinic because of gradually worsening symptoms. She was alert and oriented with no acute distress and normal vital signs. With the exception of her abdominal examination, no abnormalities were detected. Her abdominal examination was remarkable for abdomen distension with burn scars on the anterior abdominal wall in the epigastric and left hypogastric region. There were no striae, dilated veins, rashes or visible peristalsis and bowel sounds were normal. The abdomen was soft with a large, firm and fixed mass (~15 cm × 15 cm) palpated in the epigastric/left hypochondriac region. Rectal examination was unremarkable. Laboratory studies included a complete blood count, renal function test, liver function test and a coagulation profile. All were normal. A repeat CECT of abdomen and pelvis showed: a large heterogeneously enhancing exophytic lesion (size ~18 cm) abutting the pancreatic head and superior mesenteric vessels, features suggestive of an EGIST (Fig. 1d–f).

**Figure 2:**
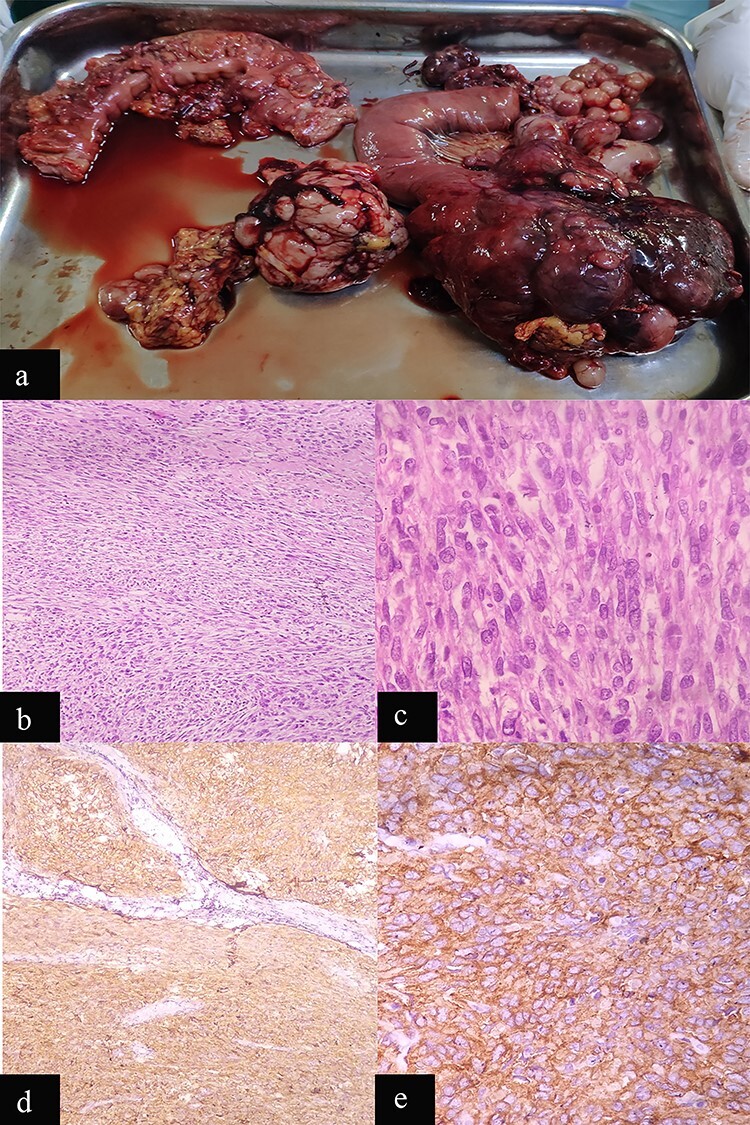
Case 1: macroscopic and microscopic features of extragastrointestinal stromal tumor. (**a**) Macroscopic appearance of tumor. (**b**) Low-power view. Tumor cells are arranged in fascicles. H & E stain. Original magnification ×100. (**c**) High-power view. The tumor cells have scant to moderate amount of cytoplasm. Nuclei are oval to elongated and have coarse nuclear chromatin. Mitosis is also noted (arrow). H & E stain. Original magnification ×400. (**d**) Low-power view. IHC showing diffuse staining by CD 117. Original magnification ×100. (**e**) High-power view. IHC showing diffuse cytoplasmic staining by CD 117. Original magnification ×400.

The patient underwent exploratory laparotomy and was found to have multiple masses on the jejunal mesentery, the largest measuring 15 cm × 14 cm with adherence to jejunal serosa, but not invading the mucosa. Another separate mesenteric nodular mass measuring 11 cm × 6 cm was found along with multiple nodules in the pericolic fat of the transverse mesocolon ranging in size from 0.5 to 2.0 cm (Fig. 2a). Part of the jejunum and the mesentery enclosing the tumor, as well as the transverse colon with mesocolon containing nodular masses was resected, then an end-to-end jejuno-jejunal and colo-colonic anastomosis was performed. The hand-sewn anastomosis was carried with the first layer closed using a full thickness vicryl suture and the second layer using a Lambert seromuscular suture. The patient tolerated oral intake from the third day of surgery.

Histopathology examination revealed a mesentery and pericolic fat GIST with a high-mitotic index (17/50 high-power field [HPF]), AJCC stage pT4N0 (Fig. 2b and c). The immunohistochemistry (IHC) was positive for c-Kit (CD117; Fig. 2d and e).

The postoperative course was uneventful with significant improvement in symptoms and the patient was discharged on the seventh postoperative day with oral Imatinib. Follow-up 7-month later showed no clinical evidence of tumor recurrence or distant metastasis when scanned with ultrasonography. This patient will continue on a plan of surveillance with ultrasonography, blood cell counts, chemistries and physical examination at 3–6-month intervals and CT or MRI of abdomen and pelvis at 1-year interval until 3 years.

## CASE PRESENTATION-2

**Figure 3:**
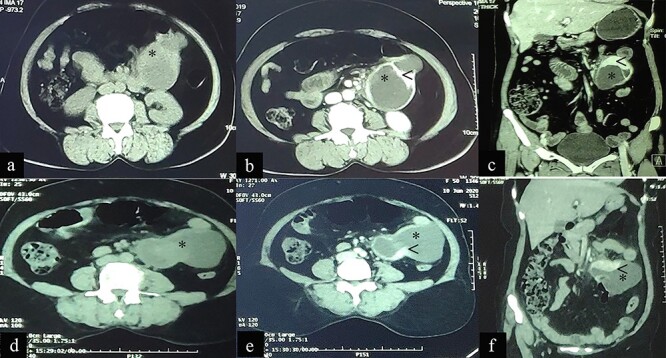
Case 2: CT imaging of Extragastrointestinal stromal tumor. (**a**) Non-contrast axial; (**b**, **c**) contrast enhanced axial and coronal reformatted CT images of abdomen demonstrates intraperitoneal cystic lesion (*) with eccentric enhancing solid component (<) abutting kidney and bowel. (**d**) Non-contrast axial; (**e**, **f**) contrast enhanced axial and coronal reformatted CT images of abdomen of same patient after 1-year interval follow up demonstrates increased size of the lesion (*).

A 50-year-old female presented with a 2-year history of intermittent left flank pain occurring once every 1–2 months with each episode lasting for 6–7 days, which sometimes required analgesics. She also complained of gradually increasing anorexia, as well as abnormal stool consistency. The patient was previously seen at another facility 12-month earlier where a CECT of the abdomen showed a cystic lesion (8.98 × 5.34 cm) with enhancing wall, septa and a solid component (28 × 13 mm) in the left abdominal cavity abutting the jejunum anteriorly, psoas muscle posteromedially and the small bowel both superiorly and inferiorly, suggestive of an EGIST (Fig. 3 a–c). She initially opted for conservative treatment but her symptoms worsened and hence she visited to our outpatient clinic for further management.

Her past medical history was significant for a 10-year history of hypertension treated with amlodipine 5-mg daily and a total abdominal hysterectomy 25-year ago for uterine fibroid.

On presentation she was alert and oriented with no acute distress and normal vital signs. Her physical examination was unremarkable except for her abdominal findings, which revealed a soft, non-distended abdomen with a lower midline surgical scar, no striae, dilated veins, rashes or visible peristalsis. Bowel sounds and rectal exam were normal. A fixed mass (12 cm × 8 cm) was appreciated in the left-upper quadrant. A complete blood count, renal function test, liver function test and coagulation profile were all normal. Repeat CECT of the abdomen and pelvis showed an intraperitoneal cystic lesion (83 × 80 × 59 mm) with an eccentric enhancing solid component (73 × 29 mm) within the mesentery abutting an area from the distal duodenum to the jejunal loop inferiorly with features suggestive of an EGIST (Fig. 3d–f).

**Figure 4:**
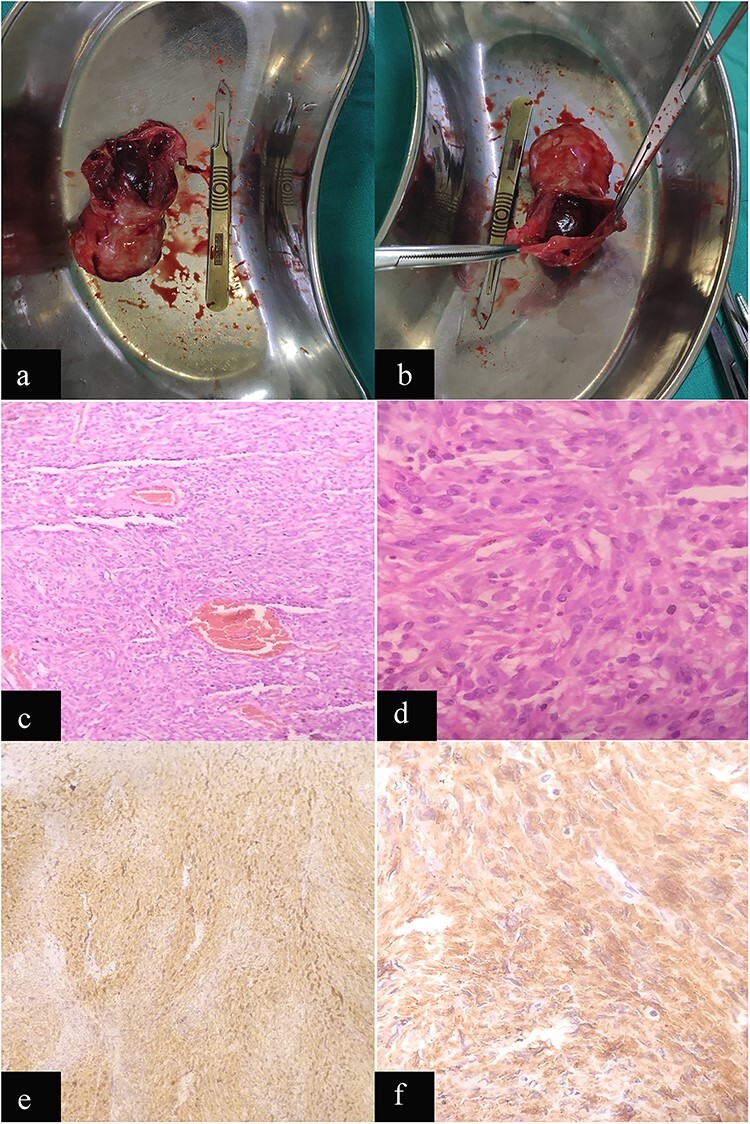
Case 2: macroscopic and microscopic features of extragastrointestinal stromal tumor. (**a**) Macroscopic appearance of tumor. (**b**) Macroscopic appearance of tumor showing ruptured cystic component. (b) Low-power view. Tumor cells are arranged in short fascicles. H & E stain. Original magnification ×100. (**c**) High-power view. The tumor cells have scant to moderate amount of cytoplasm. Nuclei are oval to elongated and have coarse nuclear chromatin. H & E stain. Original magnification ×400. (**d**) Low-power view. IHC showing diffuse staining by CD 117. Original magnification ×100. (**e**) High-power view. IHC showing diffuse cytoplasmic staining by CD 117. Original magnification ×400.

The patient underwent an exploratory laparotomy which revealed a mass in the left upper quadrant with a diameter of 16-cm originating in the proximal jejunal mesentery with a 9-cm solid component and the rest containing clear fluid. The mass was mobile with no intestinal serosal adhesion, no palpable mesenteric lymph nodes and no palpable liver nodules (Fig. 4a and b). While dissecting the mesenteric mass, the cystic part ruptured, spilling its contents intraperitoneally. The peritoneal cavity was immediately washed with a large volume of normal saline. The mass was completely excised.

Histopathologic examination revealed a spindle cell neoplasm consistent with GI tumor with mitotic index (2/50 HPF), AJCC stage pT4N0 (Fig. 4c and d) and a IHC positive for c-Kit (CD117; Fig. 4 e and f).

The postoperative course was uneventful and the patient was discharged with oral Imatinib therapy. Follow-up 3-month later showed no clinical evidence of tumor recurrence or distant metastasis when scanned with ultrasonography. The surveillance plan for this patient is also similar as the first case.

### GIST and EGIST: a short review

The mesenchymal or stromal tumors arising in the GI tract are classified as subepithelial neoplasm and broadly divided into two groups. The most common group consists of neoplasms that are collectively referred to as GISTs, whereas the less common group consists of neoplasms similar to tumors arising from soft tissue in other parts of the body [7, 8]. Mesenchymal tumors of GI tract are rare constituting only 1% of primary GI cancers [7, 8]. Data from the National Cancer Institute’s surveillance, epidemiology and end results (SEER) program suggest that GISTs predominantly occur in middle-aged and older people. Over the last 30 years, characterization of GISTs has improved beyond its original concept as spindle or epithelioid tumors arising from smooth muscle [9]. Based on their histologic characteristics, GISTs have unique morphologic and immunophenotypic profiles when compared with tumors arising from smooth muscle in other parts of body. GIST consists of heterogenous group of myogenic, neural or bidirectional differentiation or even null phenotype [2]. Although earlier detection of GIST relied upon the CD34 positivity in two-third of tumors, the true molecular characterization of GIST started after the identification of CD117 (product of c-kit protooncogene), which is overexpressed in ~90% GIST. KIT mutations are observed in ~80% GIST, whereas neither KIT mutation nor CD117 overexpression was detected in other tumors originating from mesenchymal cells [8, 10]. Most of the KIT gene mutation (~75%) are seen in exon 11 with the remaining in exon 9, 13 or 17 [11]. In 2004, another high specific marker of GIST, anoctamin 1 (also known as DOG-1), a calcium activated chloride channel, was described and found in 98% of GISTs without the association of CD117. The combination of CD117 and anoctamin 1 significantly raised the accuracy of diagnosis of GIST [12].

**Figure 5:**
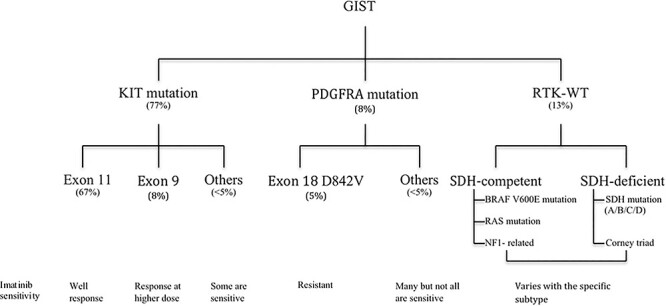
Figure: stratification of GIST based on current knowledge.

A subgroup of GIST showing negative KIT mutations, demonstrated mutations in receptor tyrosine kinase and platelet derived growth factor receptor alpha (PDGFRA). The sporadic or wild type GIST lacking mutation in KIT/PDGFRA showed either somatic or germline loss of function mutation in the subunits of succinate dehydrogenase (SDH) complex or other rare mutations [13]. Based on currently available data, GIST can be stratified as shown in Fig. 5.

Current treatment strategies for GIST are mainly guided by risk stratification based on the parameters such as tumor size, location, mitotic index and tumor proliferation rate. However, this classification system is vulnerable to over classification of malignant grade and under classification of non-gastric tumors [14]. To ameliorate this problem, additional parameters such as incomplete resection (R1), tumor rupture etc., are utilized to determine the prognosis, as well as guiding management [15, 16]. Conflicts in treatment recommendations may be the result of the incorporation of mutational status in the risk stratification and incomplete molecular characterization of GIST. Therefore, the treatment of GIST on the basis of pathological and mutational characteristics may remain the only solution at present. Complete surgical resection followed by targeted adjuvant therapy or neoadjuvant regimen followed by surgical resection remains the mainstay treatment. To date, Food and drug administration (FDA) has approved only four targeted drugs for the treatment of GIST (www.fda.gov) as shown in Table 1.

**Table 1:** List of FDA approved drugs for the treatment of GIST and their corresponding targeted molecule

**Target**	**Drugs**	**Indication**
**KIT, PDGFR, ABL**	Imatinib	First-line
**PDGFR, VEGFR1/2/3, KIT**	Sunitinb	Second-line
**KIT, PDGFR, RAF, VEGFR1/2/3**	Regorafenib	Third-line
**PDGFRA**	Avapritinib	PDGFRA exon 18 mutation including D842V mutation

As the name implies, GIST found outside the GI tract, comprising < 5% of total GIST are classified as EGIST. EGIST are typically found in the mesentery, omentum, retroperitoneum, abdominal wall, liver, pancreas, gall bladder, urinary bladder, seminal vesicle, prostate, vagina and pelvis [3].

Although EGIST are recognized as similar to GIST, several studies have noted some differences in their characteristics. Interstitial cells of cajal residing on the intestinal wall give rise to GIST whereas cajal-like cell or pluripotent stem cell located outside GI tract forms EGIST [3, 4]. Abdominal pain associated with an abdominal mass are the most common symptoms of EGIST while GI bleeding due to mucosal erosion is a rare presentation. This helps to explain why patients with EGIST normally appear at a later stage with possible metastasis at the time of diagnosis.

Contrast CT typically shows some distinctive features, such as a large abdominal mass with peripheral enhancement of unknown origin displacing neighboring structures as well as possible enlargement of abdominal lymph nodes. Whereas this presentation is typically absent in GIST [1]. CT imaging cannot completely distinguish EGIST from other abdominal pathological masses such as lymphoma, liposarcoma, leiomyosarcoma and fabrosarcoma. However, the presence of hemorrhage, calcification, necrosis or cystic changes could increase the suspicion for EGIST [17–19]. They are generally discovered incidentally during laparotomy or as part of the workup of an abdominal mass. As in GIST, pathology and IHC (positive staining for CD117) is the only confirmatory diagnosis of EGIST [3]. Some studies have promise using other immunological markers in EGIST, such as Bcl-2, CD34, smooth muscle actin, Desmin and DOG1 [3, 4].

To the authors’ knowledge, unlike GIST, there is no consensus on classification, grading and management of EGIST. Although some authors recommend similar grading and management protocols as per GIST [20], the distinguishing behavior of EGIST, such as early age onset, larger tumor at presentation, and more aggressive behavior with poorer prognosis requires that EGIST be considered as the separate entity. Even though tumor cells morphology, mitotic indices, involvement of abdominal lymph nodes and tumor necrosis are considered as prognostic factors, but more studies are required to establish the validity of these prognostic indicators [4].

The current treatment recommendation for EGIST is similar to that for GIST; en bloc resection of the tumor with pathology proven negative margins followed by adjuvant imatinib [5]. However, the efficacy of imatinib in EGIST remains controversial as it possesses different behavior from GIST [3, 4]. But, the use of imatinib is recommended for unresectable tumors with dose escalation if disease progresses. As opposed to GIST, KIT mutation at exon 11 is found in only 37.6% EGIST and this subgroup shows better response to imatinib than other EGIST groups [4].

The larger size tumor at presentation and absence of early GI symptoms in EGIST often results in a poorer prognosis of EGIST (1, 3 and 5 years overall survival rate 91.7, 61.1 and 48.9%, respectively) compared with GIST (1-, 3-, 5-year overall survival rates of 94.0, 88.1 and 82.4%, respectively) [5].

In both cases reported here, the patients had abdominal symptoms earlier and were suspected to have an EGIST, but treatment was delayed because of patient preference. The gradually increasing tumor burden produced more severe symptoms, compelling them to seek treatment. This demonstrates why EGIST patients often seek medical care only in the later stages of disease, resulting in poor outcomes. The large tumors resected in these patients are typical, as reported in other cases. However, the multiple tumors located at both proximal jejunal mesentery and the pericolic fat of the transverse mesocolon in our first case is unique. This could be due to seeding of tumor within the abdominal cavity without distant metastasis and may be a novel characteristic of a subgroup of EGIST that was not previously defined. As reported in the literatures, the diagnosis of EGIST in our cases was also confirmed with cytology and IHC positive for CD117. In addition, multiple tumors with large size, the presence of necrosis, and the high-mitotic rate (17/50 HPF) in first case and the large tumor size with spillage of fluid intraoperatively in second case suggest high-risk EGIST. Therefore, we decided to treat both patients with adjuvant imatinib. Follow-up surveillance is needed to determine the true prognosis for these patients.

To conclude, EGIST is a rare entity with unclear characteristics. A growing body of reported cases may help further our understanding of this disease. The current evidences do not adequately explain the clinicopathological features and malignant potential of EGIST. Timely diagnosis and aggressive surgical intervention remain the mainstay of treatment. The role of imatinib in EGIST as an adjuvant or neoadjuvant remains controversial. A thorough molecular characterization of EGIST, considered as separate entity from GIST, would help us better understand this disease ultimately provide a guide for optimal management resulting in better patient outcomes.
